# A video book of ophthalmic skills for medical students

**DOI:** 10.5116/ijme.5c60.0c16

**Published:** 2019-02-26

**Authors:** Kaveeta Kaur Bedi, Michael Okorie

**Affiliations:** 1 Department of Medical Education, Brighton and Sussex Medical School, UK

**Keywords:** Video book, ophthalmic skills, medical students

## Introduction

The General Medical Council sets the standards for all UK medical school curriculums to enable newly qualified doctors to achieve the required skills.^1^ Ophthalmic skills are essential and can facilitate the diagnostic process but are often neglected and given little prominence within undergraduate medical curricula. It is also recognised that ophthalmology teaching is frequently substandard and approaches widely varied.^2^ This article explores potential reasons for suboptimal student ophthalmology teaching and presents an educational resource developed at Brighton and Sussex Medical School (BSMS).

### Deficits in teaching and learning

There are a number of reasons which might explain why teaching in ophthalmology at an undergraduate level is suboptimal. Firstly, as a result of limited medical undergraduate curriculum time, the focus shifts to the acquisition of appropriate knowledge, skills and behaviour in core medicine and surgery, which relegates some subspecialties. Consequently, students are provided with a limited opportunity to obtain experience in subspecialties such as ophthalmology. Secondly, in busy healthcare delivery settings, these limited learning opportunities present a conflict between service provision and teaching.  A final contributing factor to poor teaching is that ophthalmic skills are challenging to learn, practice and become fully competent in performing, especially in a short time frame.

Eye presentations are common in medical education. Therefore, if students are better skilled in ophthalmology competencies, they might be more confident in diagnosing and managing patients outside of the specialist eye care services, such as within the emergency department. Ultimately, this might lead to more rapid and appropriate management resulting in improved patient outcomes, satisfaction and throughput. Furthermore, it might ease the demand for specialist eye services. Therefore, it is imperative that medical schools recognise and attempt to mitigate this deficit.

### An innovative approach to teaching and learning

Student feedback at BSMS has highlighted the difficulties encountered in the teaching and learning of ophthalmic skills. Besides, ophthalmologists have expressed that although there is an expectation that students should be familiar with the equipment and how to perform the skills, they lack time to teach the students anything more than the pathology. There is no single optimal approach to address this issue, but there is scope for improvement in the delivery of teaching.  More specifically, ensuring students know how to perform a skill means that they identify interesting disease conditions in the clinics.

In an attempt to fill this gap, we have developed an educational resource to aid in the teaching and learning of core ophthalmic skills at BSMS. We have created a series of video tutorials that explain and demonstrate how to perform examinations of the visual field and visual acuity, fundoscopy, assessment of pupillary light reflexes, ocular movement examination and an introduction to an ophthalmoscope.

This innovative resource is one way to bridge the skills gap, potentially, with multiple benefits. Firstly, it is an online resource that can be accessed repeatedly by all students, anywhere and at any time.  In addition, it is a standardised resource that ensures students are provided with consistent and benchmarked learning opportunities that appropriately address their learning needs. Furthermore, the production of this learning resource is at a one-off cost and is relatively inexpensive. Once produced, there are no further costs, which is a stark contrast to other modes of delivery of teaching such as practical skills sessions, which require staff time, equipment and adequate learning space. This educational resource also has the advantage that it can be used in conjunction with existing teaching resources in an expanding curriculum. Thus, students are not disadvantaged by limited clinical exposure in ophthalmology. Furthermore, the tutorials are excellent for all learner types, utilising both visual and audible mediums. Finally, this resource is innovative; it can be applied to a variety of subjects and offers new teaching styles.

Despite the apparent benefits of these teaching videos there are some limitations. Production requires the availability of and access to recording equipment, editing software as well as space to record. Secondly, the level of technical expertise required is excellent and therefore, individuals recording and editing the scenarios need to be trained to do so. Furthermore, time is required to develop these resources. This requires technical support staff, clinicians to write and edit material and educationists to monitor the content and benchmark it against the curriculum. The time to be invested by all these staff members cannot be underestimated. However, the resources have already been created by BSMS and can potentially be shared with other medical schools.

Having launched the resource, we have observed significant student usage by the number of views recorded. We predict that as the resource becomes fully embedded as a student learning resource, its use will increase further. A suggested approach to fully embedding the video book of ophthalmic skills into the curriculum is that the course administrator emails students prior to the start of their rotation in ophthalmology advising them to watch the videos in preparation.  Also, the ophthalmology course leads encourage students to watch the videos ahead of any formal teaching within their rotation. While there are other methods to increase engagement with the resource, we are starting to see a positive response to this educational tool. However, further research is required to evaluate the perceived effectiveness of the tool from both the student and teacher perspectives.

In summary, ophthalmology is an important subspecialty for medical students. However, competing curriculum priorities lead to teaching and learning challenges, which can be challenging to overcome. This video book in ophthalmic skills developed by BSMS is an innovative educational resource which utilises a technology-enhanced learning platform and might enable students to acquire the necessary knowledge and skills in basic ophthalmic practice.

### Conflict of Interest

The authors declare that they have no conflict of interest.
